# Oral Intake of Low-Molecular-Weight Collagen Peptide Improves Hydration, Elasticity, and Wrinkling in Human Skin: A Randomized, Double-Blind, Placebo-Controlled Study

**DOI:** 10.3390/nu10070826

**Published:** 2018-06-26

**Authors:** Do-Un Kim, Hee-Chul Chung, Jia Choi, Yasuo Sakai, Boo-Yong Lee

**Affiliations:** 1 Newtree, Seongnam 13207, Gyeonggi, Korea; dkim@inewtree.com (D.-U.K.); hchung@inewtree.com (H.-C.C.); 2 Department of Food Science and Biotechnology, College of Life Science, CHA University, Seongnam 13488, Gyeonggi, Korea; wldk3176@gmail.com; 3 Central Research Institute, Jellice, Sakae, Tagajo 985-0833, Japan; sakai@jellice.com

**Keywords:** fish collagen, low-molecular-weight collagen peptide, photoaging, skin hydration, skin elasticity, skin wrinkling, collagen hydrolysate, collagen tripeptide, type I collagen

## Abstract

Collagen-peptide supplementation could be an effective remedy to improve hydration, elasticity, and wrinkling in human skin. The aim of this study was to conduct a double-blind, randomized, placebo-controlled trial to clinically evaluate the effect on human skin hydration, wrinkling, and elasticity of Low-molecular-weight Collagen peptide (LMWCP) with a tripetide (Gly-X-Y) content >15% including 3% Gly-Pro-Hyp. Individuals (*n* = 64) were randomly assigned to receive either placebo or 1000 mg of LMWCP once daily for 12 weeks. Parameters of skin hydration, wrinkling, and elasticity were assessed at baseline and after 6 weeks and 12 weeks. Compared with the placebo group, skin-hydration values were significantly higher in the LMWCP group after 6 weeks and 12 weeks. After 12 weeks in the LMWCP group, visual assessment score and three parameters of skin wrinkling were significantly improved compared with the placebo group. In case of skin elasticity, one parameter out of three was significantly improved in the LMWCP group from the baseline after 12 weeks, while, compared with the placebo group, two parameters out of three in the LMWCP group were higher with significance after 12 weeks. In terms of the safety of LMWCP, none of the subjects presented adverse symptoms related to the test material during the study period. These results suggest that LMWCP can be used as a health functional food ingredient to improve human skin hydration, elasticity, and wrinkling.

## 1. Introduction

Various factors contribute to the aging of human skin. Photoaging of the skin is induced by chronic sunlight exposure, and is a form of extrinsic aging, whereas intrinsic aging arises mainly from the decline of biological function and the action of reactive oxygen species derived from cellular metabolism [1]. Although both intrinsic and extrinsic processes cause age-dependent skin alterations, changes are more prominent in photoaged skin, as evidenced by comparisons between the facial skin which has been exposed to the sun and the buttock skin which has been protected from the sun [2,3]. In the skin severely photoaged, elastic fibers are highly disorganized and deposited abundantly throughout the dermis, and in the skin mildly photoaged, loss of fibril in micro fibrils from the dermis is shown, whereas the elastic-fiber network is gradually fragmented in intrinsic aging [4,5,6,7]. Characteristics of photoaged skin include coarse wrinkles, dryness, loss of elasticity, pigmentation [1]. In the process of photoaging resulting from solar UV radiation, skin may experience loss of collagen and elastic fibers, which, along with a reduction in the synthesis of hyaluronic acid (HA), eventually leads to wrinkle formation, dryness, and loss of elasticity [1,8,9].

In the skin, collagen, elastic fibers, and HA are major structural constituents of dermal extracellular matrix (ECM) [10,11,12]. Collagen may constitute >70% of the dry weight of the normal human skin dermis [13,14]. The dermis is composed of two morphologically different layers: the adventitial dermis of thin collagenous fibers and the reticular dermis of thick, coarse collagen bundles [15]. Skin elastic fibers are composed of an inner core of crosslinked elastin with outer layers of fibril in micro fibrils [4,16]. The HA content of the dermis is much higher than that of the epidermis, and papillary dermis has significantly greater levels of HA than reticular dermis [12]. 

Preventive remedies for skin photoaging are required because of the inevitability of exposure to sunlight. Collagen has been consumed as functional dietary supplements because of its efficacy for skin health. Collagen hydrolysates or collagen peptides (CPs) are also receiving attention, with studies in vitro and in vivo investigating the properties of various CPs, including fish-collagen hydrolysates derived from type I collagen from fish skin. In hairless mice, oral administration of Low-molecular-weight Collagen peptide (LMWCP), which is a fish-derived collagen hydrolysate, promotes recovery of collagen fibers and normal elastic fibers in the skin from degraded collagen and abnormal elastic fibers caused by UVB irradiation [8]. The study showed that this treatment leads to reductions in levels of collagenases (matrix metalloproteinase (MMP)-3 and MMP-13) expression and activities of gelatinases (MMP-2 and MMP-9), thus inhibits the breakdown of dermal collagen and results in significant reductions in skin wrinkling and trans-epidermal water loss (TEWL), and increases in skin elasticity and hydration [8]. These results suggest that LMWCP affects the regeneration of collagen and elastic fibers, thereby improving skin health (barrier function, wrinkling, hydration, and elasticity). LMWCP contains 15% tripeptide which makes it differ from other CPs which rarely contain tripeptides.

In this study, a clinical trial was performed to further investigate the health benefits of LMWCP in human skin, with a daily oral dosage of 1000 mg of LMWCP for 12 weeks in 64 female volunteers aged 40–60 years and diagnosed as having photoaged skin.

## 2. Materials and Methods

### 2.1. Preparation of Test Material and Determination of Dose

The LMWCP used herein (obtained from Newtree, Seongnam, Korea) was a collagen hydrolysate obtained from the sutchi catfish’s skin (*Pangasius hypophthalmus*), with >15% tripeptide content including 3% Gly-Pro-Hyp. A 50 mL test bottle contained 1000 mg of LMWCP along with vehicle material (Table 1). The placebo had the same formulation with the identical flavor and taste as the test material, except that LMWCP was replaced by water.

In the previous animal study using hairless mice, LMWCP exerted its effects on skin wrinkling, skin hydration, TEWL, skin elasticity, collagen formation in the dermis, and the regulation of expression of MMP-3 and MMP-13 and the activities of MMP-2 and MMP-9 at the dose of 167 mg/kg b.w. and 333 mg/kg b.w. [8]. Based on the doses of efficacy in the UVB-irradiated hairless mice, human dose was calculated using the body surface area normalization method [17]: 167 mg/kg b.w. of mice was translated to 13.5 mg/kg b.w. of human; 333 mg/kg b.w. of mice to 27.0 mg/kg b.w. of human. Thus 1000 mg/day for human was chosen as a dose for human study from the translated values applied to the average adult with 60 kg body weight: between 810 mg/day and 1620 mg/day.

### 2.2. Study Design

This clinical study had a randomized, double-blind, placebo-controlled design. It was conducted according to the applicable Good Clinical Practice and the Standard Operating Procedures of Ellead Skin and Bio Research (Ellead, Seongnam, Gyeonggi-do, Korea) from 27 February 2012, to 15 June 2012. The study protocol was reviewed and approved by the institutional review board of Ellead (Project number: EL-120208047A003).

### 2.3. Study Participants

**Figure 1:**
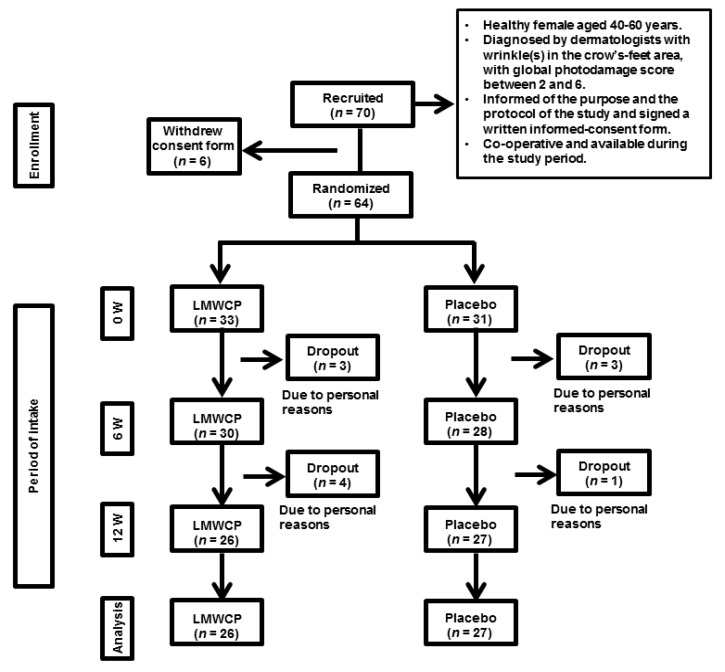
The study flow diagram. Abbreviations: LMWCP, Low-molecular-weight Collagen peptide; W, weeks.

Women aged 40–60 years (*n* = 70) who volunteered and met specified inclusion and exclusion criteria were recruited for the study (Table 2). Inclusion criteria included crow’s-feet scores between 2 and 6, as determined by dermatologists according to the global photodamage scoring system [18]. Before proceeding to the clinical study, the participants were informed clearly and precisely of the objective and the protocol of the study, and of foreseeable risks involved in the trial. Participants signed an informed-consent form. Six individuals withdrew consent, and the remaining 64 commenced the study. Eleven participants dropped out of the study for personal reasons, and 53 completed the study (Figure 1). 

### 2.4. Study Schedule

All participants took one bottle (orally) of their assigned study formulation once daily. To minimize any weakness of the study, all participants were required to refrain from intake of any similar dietary supplements, and from the use of any skincare treatments such as face masks or packs and massages. They were also not permitted to apply topical cosmetics except those provided by Ellead for a 2-week wash-out period before the study starts and for the 12-week study period, to maintain constant skin conditions. Each participant visited the research center for assessment four times in total: prior to intake of the study formulation at baseline (0 W), at 6 weeks (6 W) and 12 weeks (12 W) after intake of the study formulation, for efficacy measurements, and at 2 days after completion of intake at 12 weeks (12 W + 2 D) for safety evaluation. Prior to visiting the test facility, participants were banned from use of cosmetic products for 12 h. At each visit, participants shaved the crow’s-feet area, washed the entire face with foam cleansers, and rested for 30 min under the constant relative humidity (40–60%) and temperature (22–24 °C) prior to assessment. The same part of the face was examined at each visit, according to the same methods used in the examination conducted at 0 W. Prior to the visit for safety evaluation, participants were prohibited from eating and drinking for ≥8 h.

### 2.5. Measurement of Skin Hydration

Hydration of the skin of the cheek area of the designated side of the face is measured with a Corneometer CM 825 (Courage and Khazaka, Cologne, Germany).

### 2.6. Measurement of Skin Wrinkling

Skin wrinkling was assessed by two methods: visual assessment by dermatologists and instrumental analysis of skin-replica images. For visual assessment, cutaneous examinations of the crow’s-feet area were conducted by two dermatologists with a double-blind method based on a global photodamage scoring system [18]. The average score from the two dermatologists was used. For instrumental analysis of skin-replica images, replicas made with a silicone-based solution and a catalyst (Courage and Khazaka) were taken from the designated crow’s-feet area and analyzed with a Visiometer (SkinVisiometer SV 600, Courage and Khazaka) that evaluates the topography of the skin surface by light transmission of a very thin silicone replica based on skin-wrinkling parameters: R1 (skin roughness), R2 (maximum roughness), R3 (average roughness), R4 (smoothness depth), and R5 (arithmetic average roughness) [19,20,21].

### 2.7. Measurement of Skin Elasticity

A Cutometer MPA 580 (Courage and Khazaka) was used for assessment of skin elasticity of the designated crow’s-feet area, based on suction of the skin using a probe with negative pressure of 450 mbar, which makes the test area drawn into the aperture of the probe. Measurement was repeated three times with 2 s of suction time followed by 2 s of relaxation time for each measurement. Curves of skin deformation obtained were analyzed with Win Cutometer MPA software to obtain the values of skin-elasticity parameters: R2 (overall elasticity of the skin), R5 (net elasticity), and R7 (the ratio of elastic recovery to total deformation) [22].

### 2.8. Selection of Test Area

To retain the test area for instrumental measurements to a fixed position, a face mask made of acetate film was attached to the face after baseline measurements. The crow’s-feet area and cheek area selected as the test region were marked on the acetate film with a pen.

### 2.9. Participant Questionnaire

After 6 weeks and 12 weeks, participants filled out questionnaires regarding their subjective assessments of the efficacy of their assigned formulations and their appropriateness as a health food, safety (adverse reactions), and preference for the product.

### 2.10. Safety Assessment

Laboratory tests conducted at 0 W and 12 W + 2 D were hematological blood-chemical tests, urine tests, and vital-sign measurements. Blood-chemical test items were total protein, albumin, aspartate transaminase, alanine transaminase, γ-GTP, blood urea nitrogen, creatinine, glucose, total cholesterol, hemoglobin, hematocrit, white blood cell count, red blood cell count, platelet count, average red blood cell size (MCV), hemoglobin per red blood cell (MCH), and hemoglobin concentration per red blood cell (MCHC). Urine-evaluation parameters were urine pH, specific gravity, protein, glucose, ketone, urobilinogen, bilirubin, and nitrite. Vital signs measured were systolic and diastolic blood pressure and bodyweight. 

Safety of the test material was also evaluated by monitoring adverse reactions during the study, through information collected in interviews and questionnaires.

### 2.11. Statistical Analysis

Statistical analysis was performed in two ways: through an intention-to-treat (ITT) analysis and a per-protocol (PP) analysis, with significance level of 0.05 unless otherwise noted. Missing data were dealt with by the last-observation-carried-forward method for ITT analysis. PP analysis was used for the efficacy evaluation, and ITT analysis was used for the safety evaluation. All results in the efficacy test, blood test, urine test, and vital-signs test were analyzed using descriptive statistics and compared between before and after treatment by paired *t*-tests and between groups by independent *t*-tests. In urinalysis, for the parameters except for specific gravity and pH, McNemar’s tests and Mann–Whitney *U* tests were used after results were classified as either normal or abnormal. The incidence rate of adverse reactions in each group was compared by Chi-square tests or Fisher’s exact tests. 

## 3. Results

### 3.1. Baseline Characteristics of Participants

Participants (*n* = 64) were randomized at baseline and allocated to the test group (*n =* 33) or the placebo group (*n =* 31). Values for age, bodyweight, systolic and diastolic blood pressures, and crow’s-feet visual grade (global photodamage score) did not show any significant difference between the test group and the placebo group at baseline (Table 3).

### 3.2. Effect of LMWCP on Skin Hydration

**Figure 2:**
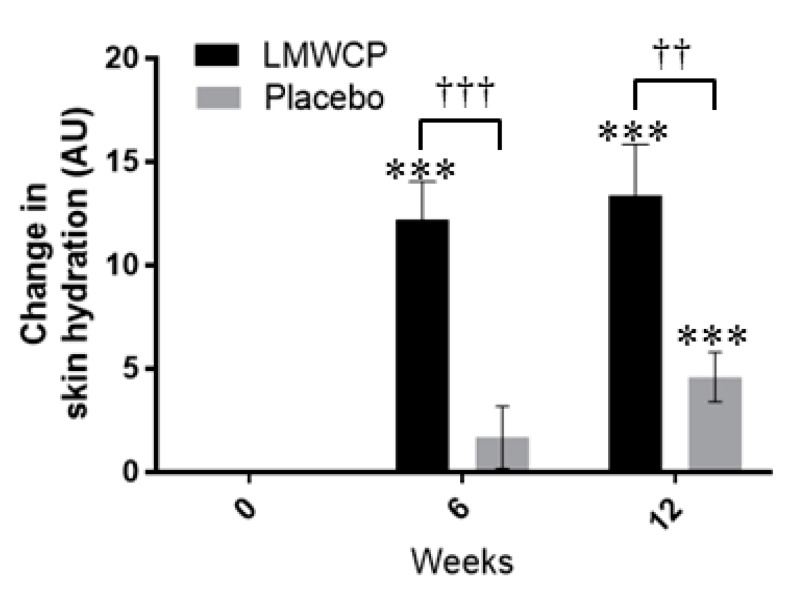
Changes in skin hydration in individuals receiving Low-molecular-weight Collagen peptide (LMWCP) preparation or placebo. Skin hydration was measured with a Corneometer CM825, and changes from baseline values are shown, with arbitrary units (AU). Data are expressed as the mean ± SEM. *** indicates a significant difference from the baseline (*p* < 0.001; *t*-test). ^††^ and ^†††^ indicate significant differences between the test group and the placebo group (*p* < 0.01 and *p* < 0.001, respectively; *t*-test).

Skin hydration in the test group was significantly greater at 6 weeks (*p =* 0.000) and 12 weeks (*p =* 0.000) than at baseline, whereas in the placebo group there was no difference from baseline at 6 weeks (*p =* 0.273), but hydration was significantly increased at 12 weeks (*p* = 0.001) (Figure 2, Table 4). In comparisons between groups, skin-hydration values were significantly higher in the test group than in the placebo group at 6 weeks and 12 weeks (*p =* 0.000 and *p* = 0.003, respectively) (Figure 2, Table 4). The increment of skin hydration in the test group was 7.23-fold greater than in the placebo group at 6 weeks, and 2.9-fold greater at 12 weeks.

### 3.3. Effect of LMWCP on Skin Wrinkling

**Figure 3:**
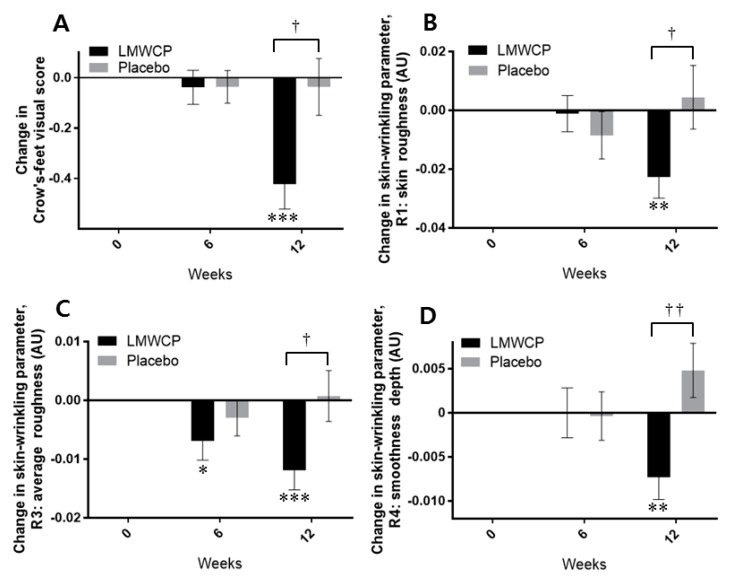
Changes in skin-wrinkling parameters in individuals receiving Low-molecular-weight Collagen peptide (LMWCP) preparation or placebo. (**A**) Changes in visually assessed Crow’s-feet scores. (**B**) Changes in skin-wrinkling parameter R1 (skin roughness). (**C**) Changes in skin-wrinkling parameter R3 (average roughness). (**D**) Changes in skin-wrinkling parameter R4 (smoothness depth). R1, R3, and R4 were measured instrumentally with a Skin Visiometer SV600. Changes in parameter values from baseline are shown in arbitrary units (AU). Data are expressed as the mean ± SEM. * *p*-values for *t*-test comparisons with baseline values; * indicates *p* < 0.05, ** indicates *p* < 0.01, *** indicates *p* < 0.001. ^†^ *p*-values for *t*-test comparisons between values in the test group and the placebo group; ^†^ indicates *p* < 0.05, ^††^ indicates *p* < 0.01.

In the test group, the visual grade related to wrinkle formation was significantly improved from the baseline value at 12 weeks (*p =* 0.000) (Figure 3A, Table 5). The test group also showed a significantly different visual grade to the placebo group at 12 weeks (*p =* 0.013) (Figure 3A, Table 5). The increment of wrinkle improvement in visual grade in the test group was 10.5-fold greater than in the placebo group at 12 weeks (Figure 3A). 

The instrumentally measured wrinkling parameters R1, R3, and R4 were significantly improved in the test group compared with the placebo group at 12 weeks (*p =* 0.043, *p* = 0.025, and *p* = 0.004, respectively) (Figure 3B–D, Table 6). In the test group, R1, R3, R4, and R5 (data not shown) were significantly improved from baseline at 12 weeks (*p* = 0.004, *p* = 0.001, *p* = 0.008, and *p* = 0.035, respectively), whereas none of these parameters was significantly improved in the placebo group (Figure 3B–D, Table 6). In the test group, R3 was also significantly improved at 6 weeks compared with baseline (*p =* 0.044) (Figure 3C, Table 6). 

### 3.4. Effect of LMWCP on Skin Elasticity

**Figure 4:**
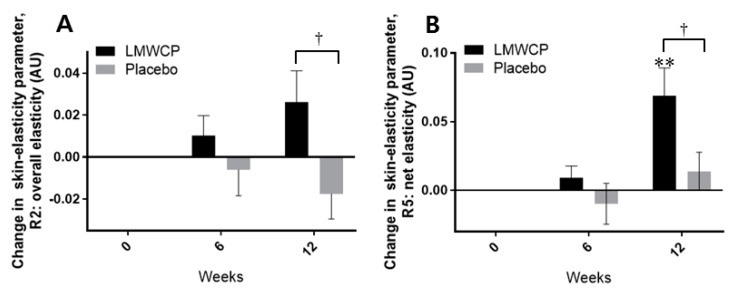
Changes in skin-elasticity parameters in individuals receiving Low-molecular-weight Collagen peptide (LMWCP) preparation or placebo. Skin elasticity was measured with a Cutometer MPA580. (**A**) Changes in skin-elasticity parameter R2 (overall elasticity). (**B**) Changes in skin-elasticity parameter R5 (net elasticity). Changes in parameter values from baseline are shown in arbitrary units (AU). Data are expressed as the mean ± SEM. For *t*-test comparisons with baseline values, ** indicates *p* < 0.01. For *t*-test comparisons between the test group and the placebo group, † indicates *p* < 0.05.

The values of the skin-elasticity parameters of overall elasticity of the skin (R2) and net elasticity of the skin (R5) were significantly higher in the test group than in the placebo group at 12 weeks (*p =* 0.025 and *p* = 0.027, respectively) (Figure 4A,B, Table 7). In the test group, however, from the baseline, only the value of R5 was significantly improved at 12 weeks (*p =* 0.002) (Figure 4B, Table 7). In the placebo group, none of the skin-elasticity parameters was shown to significantly improve from baseline values during the test period (Figure 4A,B, Table 7). 

### 3.5. Analysis of Laboratory Parameters and Adverse Reactions

The results of blood-chemical tests, urine tests, and vital-sign measurements showed that all the measured parameters of the test group and the placebo group were in normal ranges before and after intake. Statistical analysis of all the data showed no significant differences from the normal range. No adverse reactions were observed in any of the participants during the course of the study.

## 4. Discussion

The use of collagen or collagen hydrolysates as food or dietary supplements for the improvement of skin properties is increasing. LMWCP is a fish-derived type I collagen hydrolysate that is prepared by enzymatic hydrolysis and has a >15% content of tripeptides (Gly-X-Y, where X and Y are different amino acid residues that are often proline, hydroxyproline, or alanine) including 3% Gly-Pro-Hyp. The result of previous study in animal model suggested that oral intake of LMWCP may improve human skin function [8]. In this clinical trial, we investigated the efficacy of oral intake of LMWCP on human skin function. The results demonstrated that oral intake of 1 g of LMWCP once daily for 12 weeks significantly improved skin hydration, wrinkling, and elasticity in human participants.

Upon oral intake of collagen hydrolysates, small peptides derived from collagen (such as the tripeptide Gly-Pro-Hyp) can be detected in human blood, with peak concentrations observed 1–2 h after intake [23,24,25]. Gly-Pro-Hyp is readily absorbed intact across the intestine into the blood in rats [26] and humans [24]. In rats and mice, absorbed Gly-Pro-Hyp remains in the plasma for several hours, with a peak concentration ~2–4 h after administration, and can be detected in various tissues, especially in the skin, where it remains for longer than in other tissues [26]. In humans, the dipeptide Pro-Hyp (which is not digestible by peptidase) has been detected in the blood along with Gly-Pro-Hyp, suggesting that Pro-Hyp is originated from Gly-Pro-Hyp [24,25]. Transfer of biologically functional Pro-Hyp to the skin may stimulate migration and growth of fibroblasts [25,27], thereby exerting favorable effects on skin function, because dermal fibroblasts enable the synthesis of collagen, elastic fibers, and dermal HA [28,29]. Our observation of beneficial effects on the skin resulting from oral intake of LMWCP suggests that the tripeptides contained in LMWCP are efficiently absorbed and biologically active.

In this study, both the test group and the placebo group showed significantly improved skin hydration from the baseline value at 12 weeks. However, only the test group showed significant improvement earlier at 6 weeks whereas no significant improvement was seen in the placebo group. And both at 6 weeks and 12 weeks, skin hydration values were significantly higher in the test group compared with the placebo group. The improvement form baseline values in the placebo group at 12 weeks might have been due to the effect of the vehicle material and/or cosmetics supplied by investigators. However, it was proved that the skin hydrating efficacy of LMWCP alone is statistically significant after zeroing out that of the vehicle material and/or cosmetics used. Skin hydration is affected by water bounded to HA in the dermis and the epidermis [12]. The collagen-derived Hyp-containing peptide Pro-Hyp has been shown to stimulate HA synthesis by activation of *HAS2* transcription in human dermal fibroblasts in vitro [30]. It may be hypothesized that the improvements in skin hydration that occurred in the present study might therefore have resulted from increased production of HA via Pro-Hyp derived from LMWCP. However, this needs to be proved with the further studies in the future.

The improvements in skin wrinkling that we observed in this study in response to intake of LMWCP coincide well with the results of the previous animal study where expression and activation of MMPs (MMP-1(MMP-13 in rodents), MMP-2, MMP-3, and MMP-9) are down-regulated by oral administration of LMWCP in UVB-irradiated mice, thereby increasing collagen content in the skin and reducing wrinkle formation [8]. 

The mechanical properties of human skin: elasticity, resilience, and toughness, are mainly affected by collagen and elastic-fiber networks in the ECM in the dermis, and the elastic-fiber network is known to be contributing to the skin elasticity most [31]. MMP-2 and MMP-9 degrade insoluble elastin to soluble fragments [32,33], and MMP-2, MMP-9, and MMP-13 catabolize fibrillin microfibrils [34,35], so down-regulation regarding these proteins is likely to affect levels of elastic fibers in the skin [4,16]. Therefore, the oral intake of LMWCP might have favorably affected the content of both collagen and elastic fibers in the skin via down-regulation of MMPs, thereby improving skin wrinkling and skin elasticity. The skin-elasticity parameter R5 (net elasticity) represents only elastic characteristics of the skin, and so may be solely affected by elastic fibers [22]. In this study, R5 in the test group significantly improved from the baseline level at 12 weeks, while both R2 (overall elasticity) and R5 were higher with significance compared with the placebo group, suggesting that LMWCP directly exerts its effect on skin elasticity of the photoaged skin because one of the inclusion criteria for recruiting participants was to specify the eligible photodamage score of the UV-exposed site, crow’s feet. Skin hydration also strongly affects skin elasticity, because conformational change in elastin occurs only in hydrated protein [36]. The increased level of skin hydration that we observed following intake of LMWCP may therefore have contributed to the increase in skin elasticity. No adverse reactions to LMWCP were observed, and the results of laboratory tests and vital-sign tests in the LMWCP group were in normal ranges. 

Our results demonstrated that oral intake of LMWCP (1000 mg once daily for 12 weeks) efficiently improved the health of photoaged skin in a cohort of women aged 40–60 years, by significantly improving skin hydration as early as after 6 weeks of intake, and skin wrinkling and elasticity after 12 weeks of intake. The LMWCP preparation was well tolerated by participants in the study. Although this study was done only for female individuals at the age between 40 to 60 years old, the results may still be valid for the male and other age groups. However, the further studies using both male and female at different age group are recommended to generalize the anti-photoaging efficacy of LMWCP. Furthermore, it would be also desirable if the larger number of participants is used hence the more robust results can be expected in the future studies. Interest in the development of anti-skin aging agents has been increasing considerably, and LMWCP may have potential as a safe and effective ingredient for health-functional food or dietary supplements, to improve skin wrinkling, hydration, and elasticity.

## 5. Conclusions

The LMWCP is a safe health functional food ingredient with anti-skin photoaging efficacy which can effectively improve hydration, elasticity, and wrinkling in human skin at the dose of 1000 mg once daily.

## Figures and Tables

**Table 1:** Ingredients of test and placebo preparations.

Ingredients	Test		Placebo	
	Content (mg)	Content (%)	Content (mg)	Content (%)
Low-molecular-weight Collagen peptide	1000	2	0	0
Vitamin C	100	0.2	100	0.2
Fruit concentrate mix	3000	6.0	3000	6.0
Flavor mix	200	0.4	200	0.4
Excipients	1900	3.8	1900	3.8
Sweetener	12.5	0.025	12.5	0.025
Water	43,787.50	87.575	44,787.50	89.575
Total	50,000	100	50,000	100

**Table 2:** Inclusion and exclusion criteria for recruitment of participants.

**Inclusion Criteria**
Healthy female aged 40–60 years. Diagnosed by dermatologists with wrinkle(s) in the crow’s-feet area, with global photodamage score between 2 and 6. Informed of the purpose and the protocol of the study and signed a written informed-consent form. Co-operative and available during the study period.
**Exclusion Criteria**
History of allergies to cosmetics, pharmaceutical products, or foods containing ingredients included in the test formulation. Diagnosis of any systemic illness that may be aggravated by participation in the study. Use of oral retinoids or oral steroids in the 6 months prior to initiation of the study. Use of topical retinoids, anti-wrinkle cosmetic products including retinol and/or AHA, or moisture-rich cosmetic products within the 3 months prior to initiation of the study. Skincare therapy using lasers or peeling within the 3 months prior to initiation of the study. Current participation in another clinical test, or participation in any type of wrinkle study within the 3 months prior to initiation of the study. Abnormal liver function or abnormal renal function. Current smoking habit or history of smoking within the past 1 year. Excessive alcohol intake. Women who had undergone, or planned to undergo, pregnancy or breastfeeding. Blood pressure >140/90 mmHg or hypertension with intake of a diuretic. Problems with overall findings in blood-test results as determined by a specialist. History of asthma or allergic disease. History of depression, schizophrenia, alcoholism, drug addiction, or mental illness. Current or previous intake of contraceptives, female hormones, obesity drugs, absorption inhibitors, antidepressants, or appetite suppressants. Any condition judged by the investigator to be unsuitable for participation in the study.

**Table 3:** Baseline characteristics of participants.

Variable	Test Group (*n =* 33)		Placebo Group (*n =* 31)		*p*-Value ^†^
	Mean (SD)	Min, Max	Mean (SD)	Min, Max	
Age (years)	48.00 (4.50)	40, 59	48.35 (4.32)	43, 57	0.749
Weight (kg)	54.45 (5.25)	44.3, 70.5	56.73 (4.46)	48.1, 65.3	0.067
Systolic bp ^a^ (mmHg)	117.61 (13.87)	98, 140	114.52 (11.41)	95, 140	0.336
Diastolic bp ^a^ (mmHg)	71.79 (10.29)	48, 95	69.35 (9.32)	46, 91	0.326
Crow’s-feet visual grade	3.21 (0.89)	2, 6	3.19 (0.98)	2, 5	0.937

^a^ Blood pressure. ^†^ *p*-values for *t*-test comparisons between values in the test group and the placebo group. For a significant difference by *t*-test, *p* < 0.05.

**Table 4:** Skin-hydration values measurement by Corneometer CM825.

Time-Point		Test Group		Placebo Group		Test/Placebo *p*-Value ^†^
		Mean (SD)	*p*-Value *	Mean (SD)	*p*-Value *	
**Moisture**	0 W	47.79 (12.48)		48.43 (12.52)		
	6 W	60.00 (9.91)	0.000 ***	50.12 (11.33)	0.273	0.000 ^†††^
	12 W	61.14 (11.31)	0.000 ***	53.02 (13.59)	0.001 ***	0.003 ^††^

* *p*-values for *t*-test comparisons with baseline values; *** indicates *p* < 0.001. ^†^ *p*-values for *t*-test comparisons between values in the test group and the placebo group; ^††^ indicates *p* < 0.01, ^†††^ indicates *p* < 0.001.

**Table 5:** Visual assessment of crow’s feet score by dermatologists.

Evaluation Parameter	Time-Point	Test Group		Placebo Group		Test/Placebo *p*-Value ^†^
		Mean (SD)	*p*-Value *	Mean (SD)	*p*-Value *	
**Crow’s feet** **Visual grade**	0 W	3.23 (0.95)		3.19 (1.00)		
	6 W	3.19 (1.02)	0.574	3.15 (1.03)	0.574	0.988
	12 W	2.81 (0.85)	0.000 ***	3.15 (1.03)	0.746	0.013^†^

* *p*-values for *t-*test comparisons with baseline values; *** indicates *p* <0.001. ^†^ *p*-values for *t-*test comparisons between values in the test group and the placebo group; ^†^ indicates *p* < 0.05.

**Table 6:** Skin-wrinkling parameters measured by Skin Visiometer SV600.

Evaluation Parameter	Time-Point	Test Group		Placebo Group		Test/Placebo *p*-Value ^†^
		Mean (SD)	*p*-Value *	Mean (SD)	*p*-Value *	
**R1**	0 W	0.42 (0.09)		0.43 (0.08)		
	6 W	0.42 (0.08)	0.854	0.42 (0.07)	0.301	0.475
	12 W	0.40 (0.07)	0.004 **	0.43 (0.09)	0.685	0.043^†^
**R2**	0 W	0.28 (0.04)		0.28 (0.04)		
	6 W	0.27 (0.04)	0.066	0.28 (0.04)	0.104	0.802
	12 W	0.27 (0.04)	0.055	0.28 (0.04)	0.485	0.391
**R3**	0 W	0.19 (0.03)		0.19 (0.02)		
	6 W	0.19 (0.03)	0.044 *	0.19 (0.03)	0.349	0.384
	12 W	0.18 (0.02)	0.001 ***	0.20 (0.03)	0.866	0.025 ^†^
**R4**	0 W	0.07 (0.02)		0.07 (0.02)		
	6 W	0.07 (0.02)	1.000	0.07 (0.02)	0.894	0.926
	12 W	0.06 (0.02)	0.008 **	0.07 (0.02)	0.130	0.004 ^††^
**R5**	0 W	0.18 (0.05)		0.18 (0.05)		
	6 W	0.18 (0.05)	0.519	0.18 (0.04)	1.000	0.690
	12 W	0.17 (0.04)	0.035 *	0.19 (0.05)	0.388	0.053

Skin-wrinkling parameters: R1, skin roughness; R2, maximum roughness; R3, average roughness; R4, smoothness depth; R5, arithmetic average roughness. * *p*-values for *t-*test comparisons with baseline values; * indicates *p* < 0.05, ** indicates *p* < 0.01, *** indicates *p* < 0.001. ^†^ *p*-values for *t-*test comparisons between values in the test group and the placebo group; ^†^ indicates *p* < 0.05, ^††^ indicates *p* < 0.01.

**Table 7:** Skin elasticity measured by Cutometer MPA580.

Evaluation Parameter	Time-Point	Test Group		Placebo Group		Test/Placebo *p*-Value ^†^
		Mean (SD)	*p*-Value *	Mean (SD)	*p*-Value *	
**R2**	0 W	0.74 (0.07)		0.75 (0.07)		
	6 W	0.75 (0.08)	0.287	0.74 (0.06)	0.638	0.261
	12 W	0.76 (0.06)	0.088	0.73 (0.09)	0.156	0.025 ^†^
**R5**	0 W	0.64 (0.14)		0.63 (0.13)		
	6 W	0.65 (0.15)	0.293	0.62 (0.13)	0.517	0.262
	12 W	0.71 (0.16)	0.002 **	0.64 (0.15)	0.328	0.027 ^†^
**R7**	0 W	0.36 (0.05)		0.36 (0.05)		
	6 W	0.37 (0.05)	0.722	0.35 (0.04)	0.409	0.368
	12 W	0.38 (0.04)	0.149	0.35 (0.06)	0.452	0.139

Skin-elasticity parameters: R2, overall elasticity; R5, net elasticity; R7, ratio of elastic recovery to total deformation. * *p*-values for *t-*test comparisons with baseline values; ** indicates *p* < 0.01. ^†^ *p*-values for *t*-test comparisons between values in the test group and the placebo group; ^†^ indicates *p* < 0.05.
